# Genomic signatures of artificial selection in the Pacific oyster, *Crassostrea gigas*

**DOI:** 10.1111/eva.13286

**Published:** 2021-09-02

**Authors:** Boyang Hu, Yuan Tian, Qi Li, Shikai Liu

**Affiliations:** ^1^ Key Laboratory of Mariculture (Ocean University of China) Ministry of Education, and College of Fisheries Ocean University of China Qingdao China; ^2^ Laboratory for Marine Fisheries Science and Food Production Processes Qingdao National Laboratory for Marine Science and Technology Qingdao China

**Keywords:** *Crassostrea gigas*, growth, population divergence, selection signature

## Abstract

The Pacific oyster, *Crassostrea gigas*, is an important aquaculture shellfish around the world with great economic and ecological value. Selective breeding programs have been carried out globally to improve production and performance traits, while genomic signatures of artificial selection remain largely unexplored. In China, we performed selective breeding of *C*. *gigas* for over a decade, leading to production of several fast‐growing strains. In the present study, we conducted whole‐genome resequencing of 20 oysters from two fast‐growing strains that have been successively selected for 10 generations, and 20 oysters from the two corresponding wild populations. Sequencing depth of >10× was achieved for each sample, leading to identification of over 12.20 million SNPs. The population structures investigated with three independent methods (principal component analysis, phylogenetic tree, and structure) suggested distinct patterns among selected and wild oyster populations. Assessment of the linkage disequilibrium (LD) decay clearly indicated the changes in genetic diversity during selection. Fixation index (*F*_st_) combined with cross‐population composite likelihood ratio (XP‐CLR) allowed for identification of 768 and 664 selective sweeps (encompassing 1042 and 872 genes) tightly linked to selection in the two fast‐growing strains. KEGG enrichment and functional analyses revealed that 33 genes are important for growth regulation, which act as key components of various signaling pathways with close connection and further take part in regulating the process of cell cycle. This work provides valuable information for the understanding of genomic signatures for long‐term selective breeding and will also be important for growth study and genome‐assisted breeding of the Pacific oyster in the future.

## 1 INTRODUCTION

The Pacific oyster (*Crassostrea gigas*) is an economically important aquaculture species, which has been widely farmed in the world. The global aquaculture production of Pacific oyster was up to 573,000 tons, representing the highest production in farmed animals in the world (http://www.fao.org). As the top oyster‐production country, China produces more than 80% of Pacific oysters globally (Botta et al., 2020). The success of Pacific oyster aquaculture in China is largely attributed to the selective breeding program dated back a decade ago. Since 2007, we have initiated a selective breeding program for genetic improvement of growth in Pacific oyster. Several fast‐growing oyster stains have been produced through successive selection of wild oysters collected from diverse geographic locations in China, Japan, and South Korea (Zhang et al., 2019), and superior growth performance has been achieved after generations of artificial selection (Li et al., 2011; Zhang et al., 2019).

Improvement in growth rate has been regarded as the major focus of breeding programs for most organisms. The fast‐growing oyster strains are not only valuable for the oyster industry, but also provide ideal research materials to investigate the molecular mechanisms underlying growth. Several effective approaches have been applied toward uncovering molecular mechanisms related to growth regulation. Association analysis of growth traits with SNPs allowed identifying a couple of SNP markers with significant differences in allele frequencies between fast‐growing strain and the commercial control population (Wang & Li, 2017). Comparative transcriptome analysis revealed more than one thousand differentially expressed genes between fast‐growing oysters and wild controls (Zhang et al., 2019). However, genomic signatures of artificial selection left in oyster genome remain largely unexplored. A high‐resolution genomic variation map is essential for a better understanding of how oyster genome was shaped during generations of selection.

With the rapid development of high‐throughput sequencing technologies, whole‐genome resequencing has become an effective approach to identify genome‐wide variations and detect genomic signatures of domestication or artificial selection (Rubin et al., 2010; Stratton, 2008; Xia et al., 2015; Zhang et al., 2018). So far, it has been extensively applied in a large number of plants and animals, such as maize (*Zea mays* ssp. mays L.) (Wang et al., 2020), rice (*Oryza sativa* L.) (Yano et al., 2016), chicken (*Gallus gallus*) (Rubin et al., 2010), sheep (*Ovis aries*) (Li et al., 2020), Atlantic salmon (*Salmo salar*) (Bertolotti et al., 2020), large yellow croaker (*Larimichthys crocea*) (Kon et al., 2021), and black tiger shrimp (*Penaeus monodon*) (Wong et al., 2020). In the present study, in order to profile genome signatures of selection in Pacific oyster, we performed whole‐genome resequencing of 20 oysters from two fast‐growing strains and 20 oysters from their corresponding wild populations. Fixation index (*F*_st_) and cross‐population composite likelihood ratio (XP‐CLR) approaches were combined to identify selection signatures during breeding process. This study provides valuable information for understanding of genomic signatures for long‐term selective breeding and will also be important for growth study and genome‐assisted breeding of Pacific oyster.

## 2 MATERIAL AND METHODS

### 2.1 Ethics statement

Animal experiments were conducted in accordance with the guidelines and approval of the respective Animal Research and Ethics Committees of Ocean University of China (Permit Number: 20141201). The field studies did not involve any endangered or protected species.

### 2.2 Experiment animals

**FIGURE 1:**
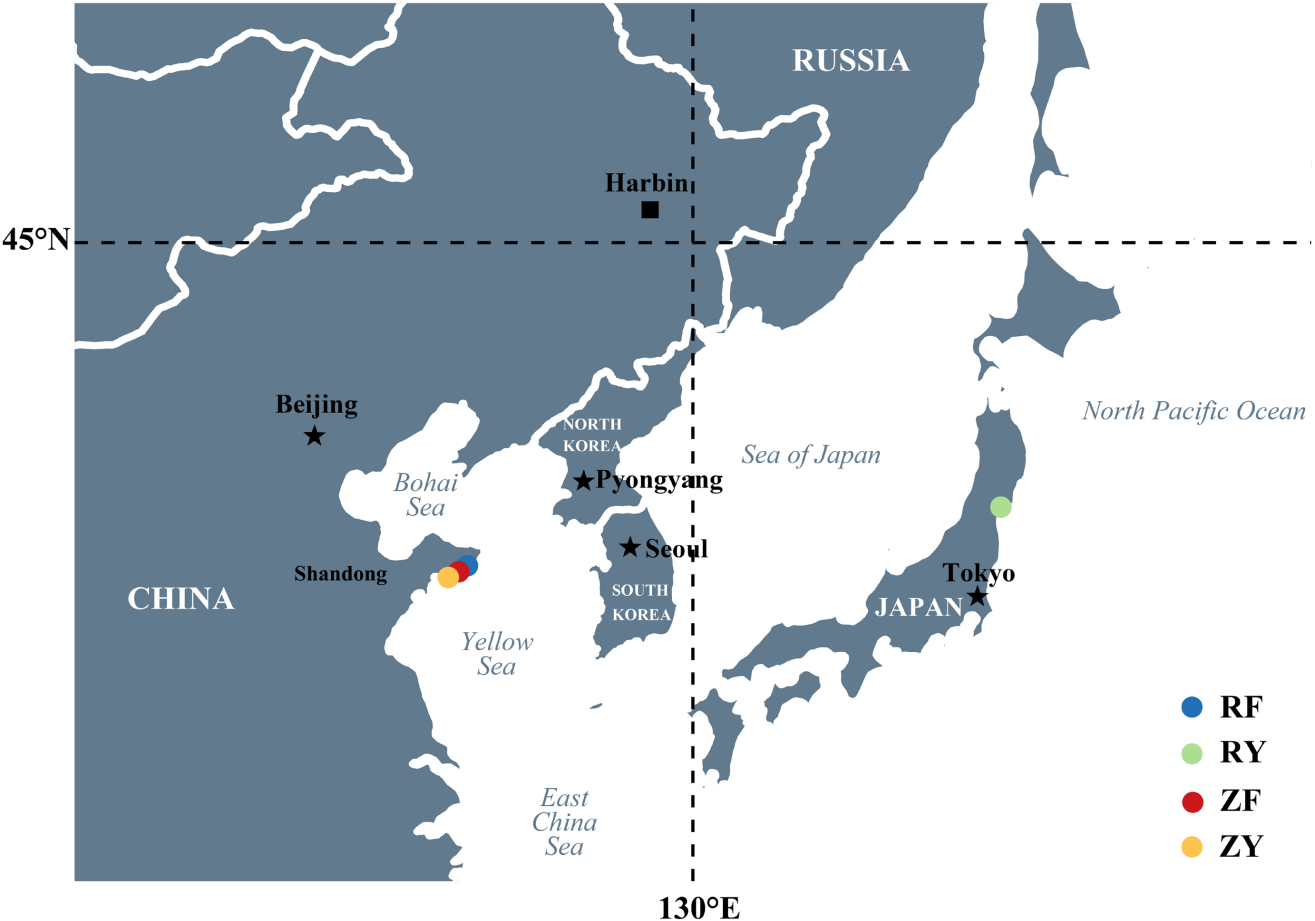
Geographic location of sampled Pacific oysters. Each dot of a given color on the East Asian map represented the geographic location of sampled Pacific oysters

**FIGURE 2:**
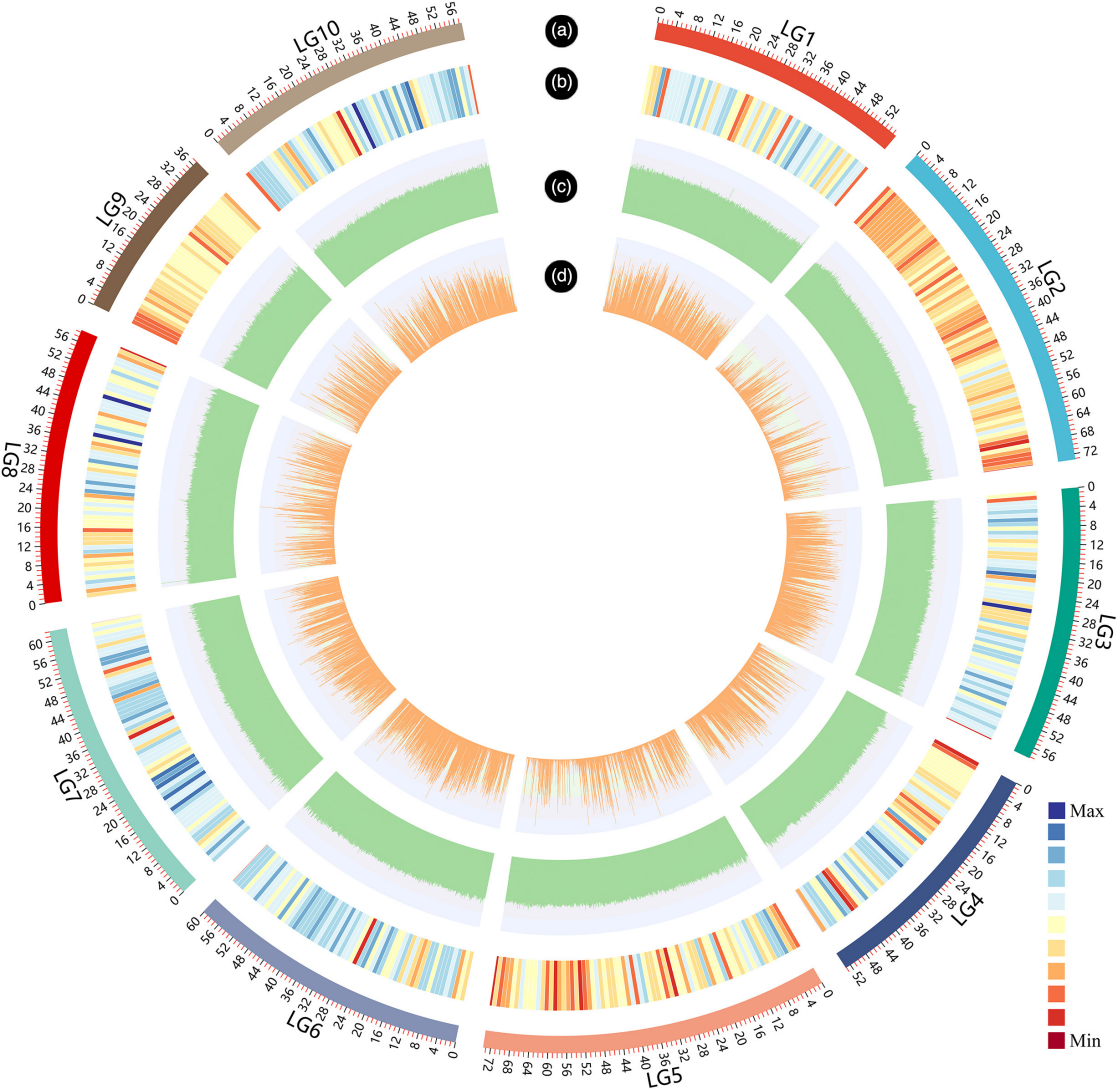
CIRCOS visualization of SNP distribution at the genome‐wide level. (a) Karyotypes of oyster genome. (b) Gene density of oyster genome. Gene numbers were calculated within 1 Mb nonoverlapping window on each chromosome. (c) GC content. (d) SNP distribution of fast‐growing RF, ZF strains and wild RY ZF populations. The bins of GC content and SNP distribution were set as 100 kb

Samples used in this study were from a selective breeding program that has been carried out for over a decade (Li et al., 2011). In brief, the oyster breeding program was initiated for genetic improvement of growth rate in 2007. Two of the selectively bred strains, denoted as RF and ZF, were produced from the breeding base populations constructed with wild Pacific oysters collected from Onagawa Bay, Miyagi, Japan (denoted as RY, 38.3°N, 141.3°E), and Rushan Bay, Shandong, China (denoted as ZY, 36.4°N, 121.3°E), respectively (Figure 1, 2). In 2017, the fast‐growing RF and ZF strains have been successively selected for 10 generations and showed superior growth performance.

### 2.3 Sample collection, DNA extraction, and sequencing

Ten individuals of each fast‐growing strain (RF and ZF) and their corresponding wild populations (RY and ZY) were collected and dissected for adductor muscle tissues. Genomic DNA was isolated from adductor muscle tissues following a modified phenol‐chloroform protocol, as described in the previous study (Li et al., 2006). DNA integrity was assessed using 1% agarose gel electrophoresis, and DNA quantity was determined by NanoDrop 2000 spectrophotometer (Thermo Scientific). A total of 40 DNA libraries with an insert size of ~350 bp were constructed, followed by sequencing on Illumina HiSeq X Ten platform (Illumina) for 150 bp paired‐end reads.

### 2.4 Read mapping and SNP genotyping

To obtain clean reads, residual adapter sequences and low‐quality regions were removed from raw reads using fastp v0.20.1 software with default parameters, followed by a second round of quality control using FastQC v0.11.9. The qualified reads were then mapped to cgigas_uk_roslin_v1 reference genome (assembly accession: GCF_902806645.1) (Peñaloza et al., 2021) using BWA mem v0.7.17. Mapping results were sorted and converted into BAM format using SAMtools v1.11. To better understand the genomic variations, SNPs were detected from fast‐growing strains (RF and ZF) and their wild populations (RY and ZY) using standard Genome Analysis Toolkit v4.1.9.0 (GATK4) pipeline. MarkDuplicates protocol of GATK4 was used to mark the duplicate reads derived from PCR amplification. FixMateInformation protocol was used to ensure that all mate‐pair information was in sync between each read and its mate pair. SNP genotypes of each oyster were estimated via local de novo assembly of haplotypes in an active region using HaplotypeCaller protocol and combined using CombineGVCFs protocol. To minimize the false positives, hard filtering of SNPs was carried out using VariantFiltration protocol with the parameters as follow: QUAL <50, QD <2.0, MQ <40.0, FS >60.0, SOR >3.0, MQRankSum < −12.5, and ReadPosRankSum < −8.0. Low‐quality SNPs were re‐filtered using vcftools v0.1.16. SNPs were filtered if their alleles were more than two, genotype quality scores were lower than 200, missing genotypes were more than 20%, minor allele frequencies were less than 5%, or mean depth coverage was less than half or greater than twice the average coverage. The qualified SNPs were annotated using SnpEff v5.0 software.

### 2.5 Population structure and LD decay analysis

Before analysis, the qualified SNPs were pruned using Plink v1.90 software with parameters of “‐indep‐pairwise 50 5 0.2” to mitigate the possible effect of LD and obtain unlinked sites. Based on pruned dataset, population structure was inferred via an expectation maximization of ADMIXTURE v1.23 software. The number of assumed genetic clusters *K* ranging from 2 to 10 with 10,000 iterations was calculated. Principal component analysis (PCA) was conducted using GCTA v1.26.0. PC1 and PC2 were plotted using ggplot2 package in R v4.0.2. Phylogenetic tree was constructed using neighbor‐joining (NJ) method implemented in VCF‐kit software and was visualized from the distance matrix with FigTree v1.4.4 software. Based on the basis of squared correlation coefficient (*r* ^2^), LD decay of each group was calculated independently for pairwise markers in a 500‐kb window using popLDdecay v3.41 software. The extent of LD decay was measured as the chromosomal distance at which the average *r* ^2^ dropped to 0.2.

### 2.6 Detection of genome selection signatures

The *F*_st_ and XP‐CLR statistical methods were used to detect genomic region under selection during the process of artificial selective breeding. As the measure of genetic differentiation between populations, *F*_st_ values were computed in 10 kb sliding windows along the chromosomes using vcftools v0.1.16 software. Windows with SNPs number less than 10 were not considered. The XP‐CLR method could accurately detect selection sweeps based on modeling the likelihood of multi‐locus allele frequency differentiation between two populations (Chen et al., 2010; Hufford et al., 2012). Nonoverlapping sliding windows of 10 kb across the chromosomes were used for scanning of selective sweeps. Minimum and maximum number of SNPs within each window were set as 10 and 200, respectively. The average recombination rate was estimated as 1.69763e‐06 cM/bp according to reference genome size and previous genetic map of Pacific oyster (Gutierrez et al., 2018). The empirical cutoffs for the genomic windows with top 1% *F*_st_ and XP‐CLR values across the whole genome were considered as selective sweeps.

### 2.7 RNA‐Seq and enrichment analysis

The previous RNA‐Seq data of ZF and ZY populations were retrieved from NCBI SRA database with the BioProject accession number of PRJNA524442 (Zhang et al., 2019). Raw reads were assessed using FastQC software and trimmed to remove low‐quality reads and obtain high‐quality reads using fastp software. The high‐quality reads were mapped to cgigas_uk_roslin_v1 reference genome using hisat2 v2.2.1 software with default parameters. The counts of reads mapped to each gene were obtained using HTSeq v0.9.1. Differentially expressed genes (DEGs) of oysters from ZF and ZY populations were determined using DESeq2 v1.32.0 R package with the threshold of |log_2_ fold‐change|≥1 and FDR < 0.05. All the coding genes of Pacific oyster were annotated using eggNOG‐mapper software (Huerta‐Cepas et al., 2017) and used for the background of the following KEGG enrichment analysis. KEGG enrichment analysis was conducted using clusterProfiler R packages (Yu et al., 2012).

## 3 RESULTS

### 3.1 Identification of genomic variations

Whole‐genome resequencing of 40 oysters, with 10 individuals from each of the fast‐growing strains (RF group and ZF group hereafter) and 10 individuals from each of their corresponding wild populations (RY group and ZY group hereafter). A total of 1813.55 million 150‐bp paired‐end reads were obtained with an average depth of ~10.26× (Table S1). Totally, 12.20 million SNPs were identified from the 40 samples after the application of stringent quality criteria, with the average SNP density of 20.71 SNPs/kb across the genome. Among which, 7,494,523 (61.39%) SNPs were located in the genic regions, including 1,266,880 (10.38%) in exons, 5,498,655 (45.04%) in introns, 167,532 (1.37%) in 5’ UTRs, and 561,456 (4.60%) in 3’ UTRs. It was obvious that variations occurring in intronic regions were more than fourfold higher than those in exonic regions. Within exonic regions, nonsynonymous SNPs (493,231, 38.93%) were less frequent than synonymous SNPs (773,649, 61.07%) with a ratio of nonsynonymous‐to‐synonymous substitutions being 0.64. The detailed information of the SNPs was provided in Table S2. Additionally, the number of SNPs in chromosomes ranged from 726,187 to 1,523,035 and the density were between 11.77/kb and 24.93/kb. As shown in Figure 2, the distribution of SNPs was closely associated with the number of functional genes across the genome (Table S3).

### 3.2 Analysis of population structure

**FIGURE 3:**
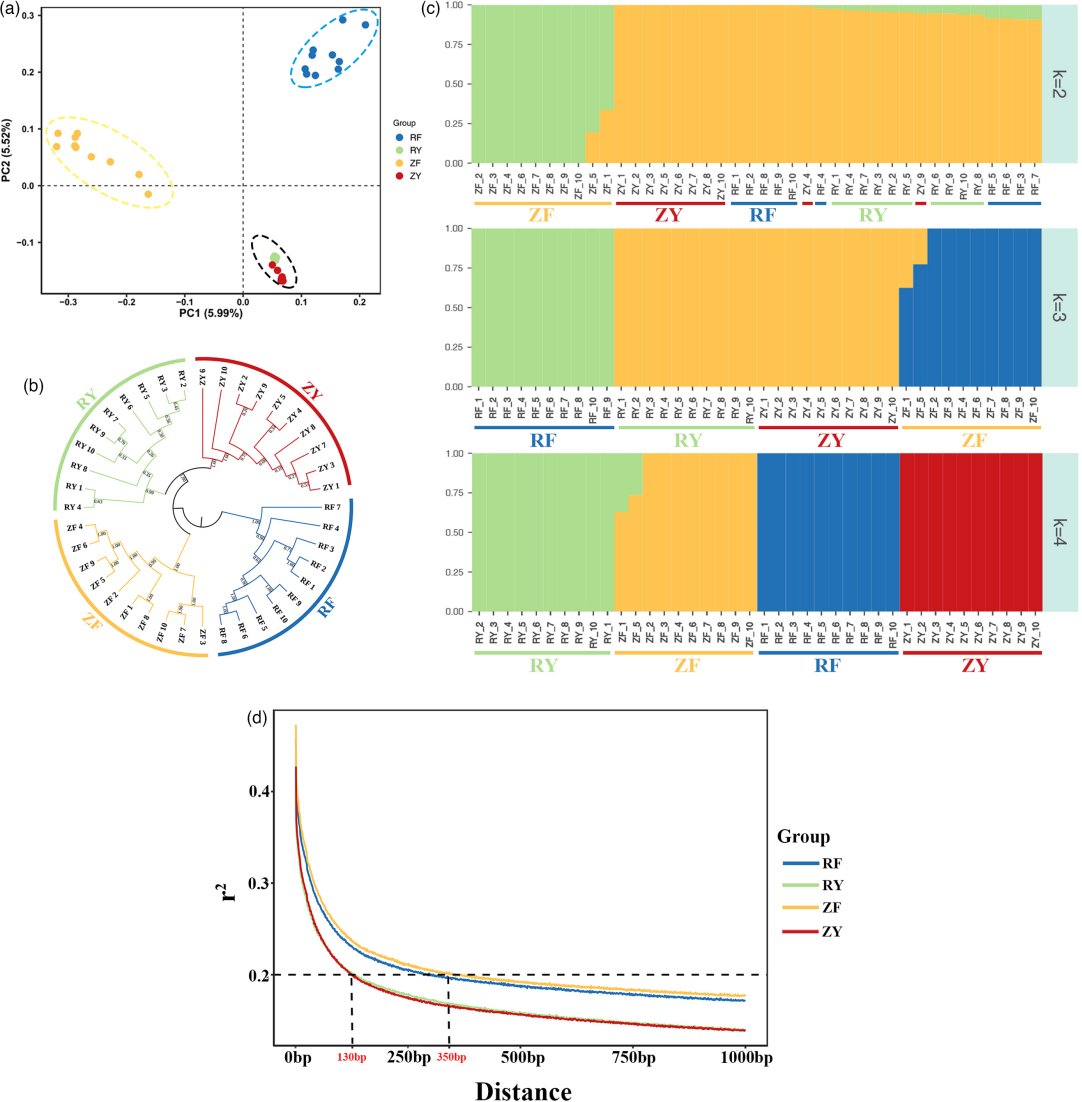
Population structure of 40 Pacific oysters. (a) Principal component analysis (PCA) of the top two components (PC1 and PC2) of 40 Pacific oysters. (b) Neighbor‐joining phylogenetic tree of 40 Pacific oysters. (c) Population structure analysis of 40 oysters with the number of kinship (*K*) ranging from 2 to 4. Each vertical bar was one Pacific oyster individual and colors represented the putative ancestral background. (d) Genome‐wide linkage disequilibrium (LD) decay in fast‐growing RF, ZF strains and wild RY, ZY populations

Population structure of the four groups of oysters was inferred using the 1,856,472 LD‐pruned SNPs (Figure 3). As shown in Figure 3a, PCA analysis classified three distinct clusters for the fast‐growing RF group, fast‐growing ZF group, and the wild groups (RY and ZY). Surprisingly, the RY and ZY groups from wild populations were clustered closely, though distinct population structures existed between the two populations (Figure S1). Great differentiations and distances were observed between the fast‐growing RF group and ZF group, which have been intensively selected for 10 generations for fast growth. Additionally, phylogenetic relationships provided strong support for the subdivision of 40 oysters into four distinct groups, with RY and ZY groups being more closely clustered (Figure 3b). An alternative view of population stratification inferred from population clustering program ADMIXTURE with *K* value ranging from 2 to 4 suggested that individuals from RY and ZY were clustered together in an admixed group when *K* = 3, while were separated into distinct groups when *K* = 4 (Figure 3c). This was largely consistent with the results of PCA and phylogenetic relationships.

The extent of LD decay was measured as the chromosomal distance when average *r* ^2^ dropped to 0.2. As shown in Figure 3d, the RY and ZY groups with wild population background clearly exhibited lower LD than the fast‐growing RF and ZF groups. LD of wild groups declined rapidly below *r* ^2^ = 0.2 at ~0.13 kb, while LD of the fast‐growing groups dropped to *r* ^2^ = 0.2 at ~0.35 kb (Figure 3d).

### 3.3 Detection of genome selection signatures

**FIGURE 4:**
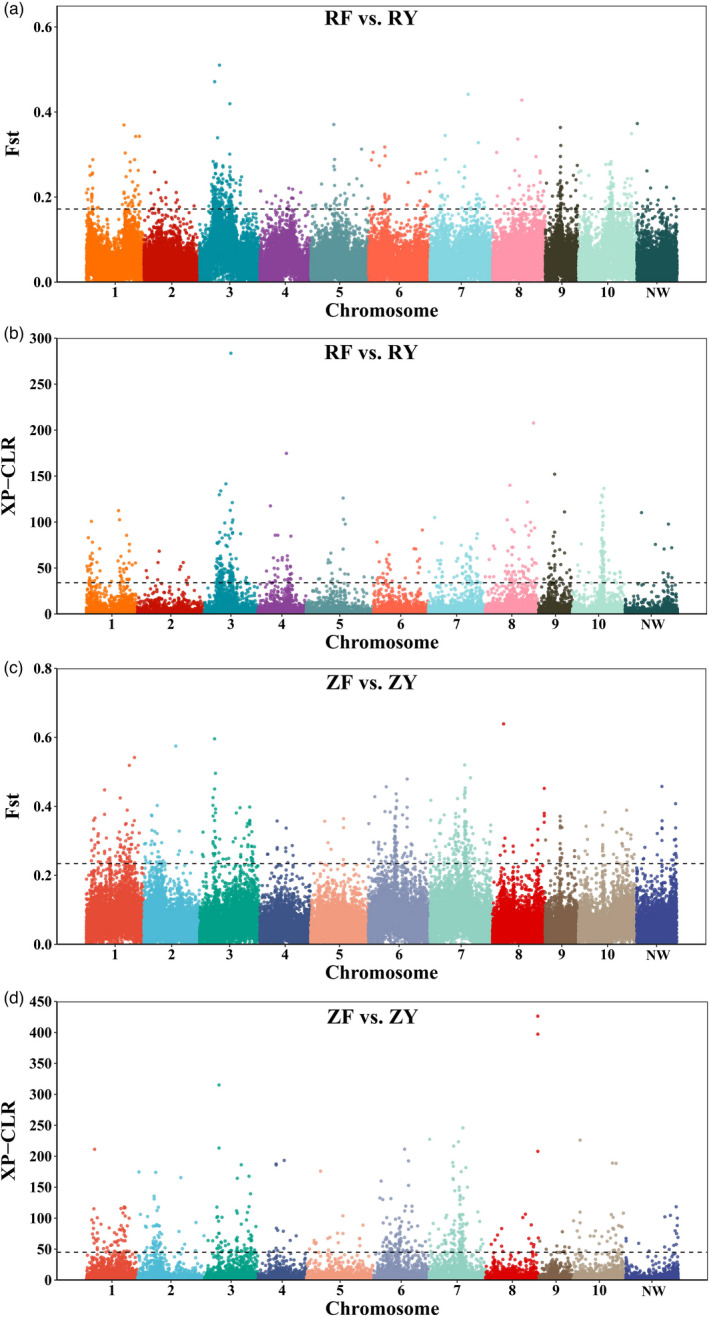
Genome‐wide distribution of selective sweeps in Pacific oyster. (a, b) Selective sweeps were identified by *F*_st_ (a) and XP‐CLR (b) in the comparison between fast‐growing RF strain and its corresponding wild RY population. The horizontal dash line represented the top 1% threshold in *F*_st_ value (0.17) and XP‐CLR scores (33.98). (c, d) Selective sweeps were identified by *F*_st_ (c) and XP‐CLR (d) in the comparison between fast‐growing ZF strain and its corresponding wild ZY population. The horizontal dash line represented the top 1% threshold in *F*_st_ value (0.23) and XP‐CLR scores (44.96)

The *F*_st_ and XP‐CLR tests were performed to scan for genomic regions with high genetic differentiation. As distinct population structures existed among two fast‐growing RF and ZF groups and two wild RY and ZY groups, we compared RF with RY (RF vs. RY) and compared ZF with ZY (ZF vs. ZY), respectively. The top 1% of the genomic windows with high values of *F*_st_ and XP‐CLR were identified as regions potentially associated with selective sweeps. Consistent with the observations in population structure analyses, distinct signatures of selection between RF vs. RY and ZF vs. ZY were observed as shown in Figure 4.

In RF vs. RY, the *F*_st_ analysis revealed a total of 423 potential selective sweeps (5.25 Mb) encompassing 570 genes (Figure 4a, Table S4), and the XP‐CLR analysis identified 434 potential selective sweeps with a cumulative size of 4.99 Mb (containing 616 genes) (Figure 4b, Table S5). Among the genes identified in RF vs. RY, 98 potential selective sweeps (1.13 Mb) and 123 genes were identified by both *F*_st_ and XP‐CLR methods. In ZF vs. ZY, a total of 393 and 446 potential selective sweeps, covering 5.24 Mb and 4.98 Mb, and harboring 499 and 537 genes, were identified by *F*_st_ and XP‐CLR, respectively (Figure 4c,d, Tables S4 and S5). Among the genes identified, 130 potential selective sweeps (1.53 Mb) and 149 genes were identified with both *F*_st_ and XP‐CLR. Combining the genes identified from *F*_st_ and XP‐CLR, a total of 1042 and 872 genes were obtained from 768 (9.23 Mb) and 664 (8.69 Mb) potential selective sweeps under artificial selection in RF vs. RY and ZF vs. ZY, respectively.

The Venn diagram of genomic regions and SNPs associated with potential selective sweeps between the separate fast‐growing populations were provided in Figure S2. A genomic region of 0.47 Mb, containing 9,747 SNPs, was identified both in RF vs. RY and ZF vs. ZY. One gene, insulin‐like peptide receptor (INR), was identified by both *F*_st_ and XP‐CLR tests in both RF vs. RY and ZF vs. ZY comparisons. Of the genes under selection in ZF vs. ZY, we found that a total of 77 genes were differentially expressed between the two populations based on the reanalysis of RNA‐seq data from a previous study (Table S6). Furthermore, we performed a direct comparison between RF and ZF populations using *F*_st_ and XP‐CLR tests to determine the genomic region that were different between the two populations. We found a large number of genomic regions with great genetic differentiation between RF and ZF populations, which provided additional evidence for their divergence (Figure S3).

### 3.4 KEGG enrichment of candidate genes under selection

**FIGURE 5:**
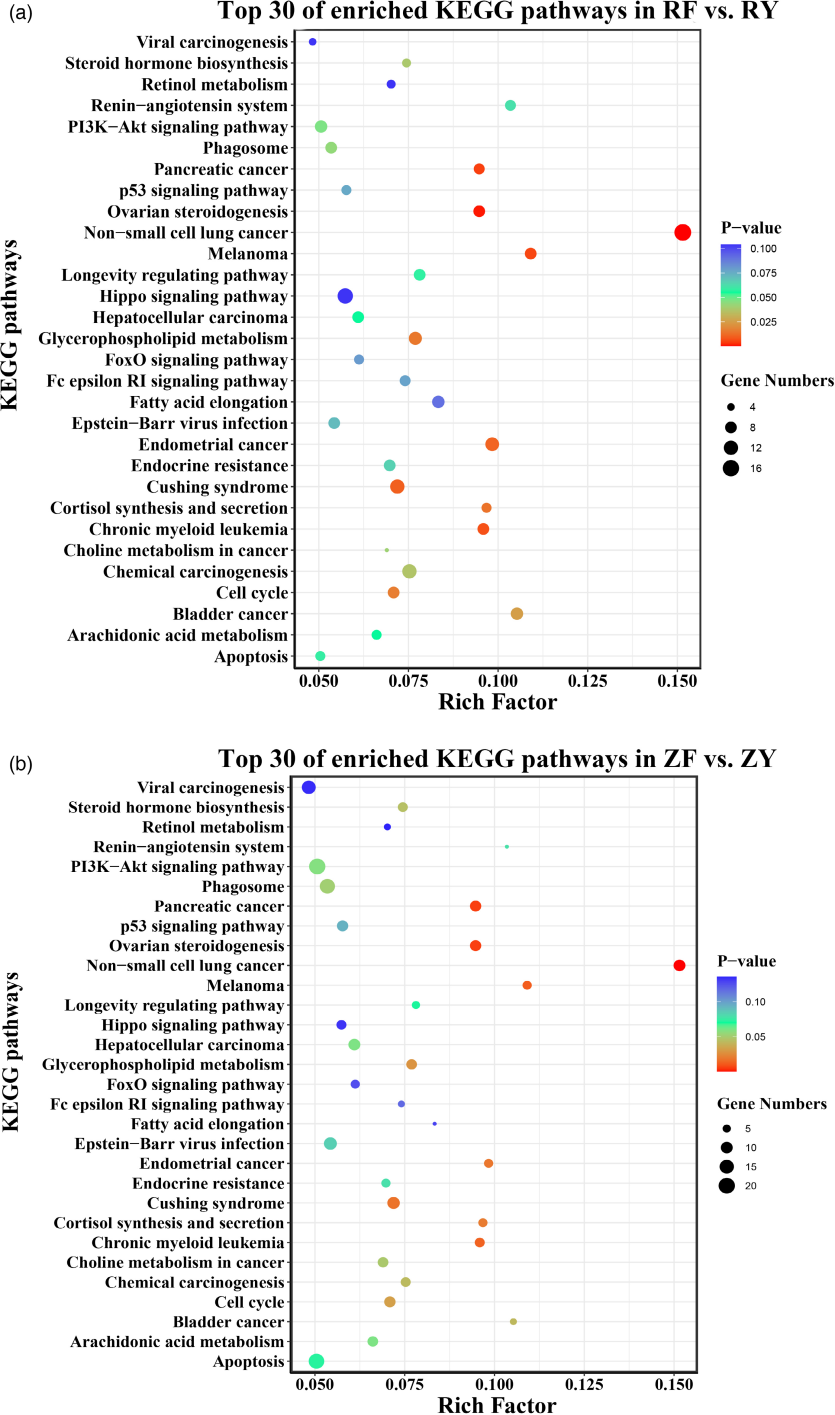
Enriched KEGG pathways of genes identified from genomic regions under selection. (a) Top 30 of enriched KEGG pathways of candidate genes identified through the comparison between fast‐growing RF strain and its corresponding wild RY population. (b) Top 30 of enriched KEGG pathways of candidate genes identified through the comparison between fast‐growing ZF strain and its corresponding wild ZY population

KEGG enrichment analysis of candidate genes identified by *F*_st_ and XP‐CLR provided the top 30 enriched KEGG pathway, ranked by *p*‐value (Figure 5, Table S7). The most significantly enriched KEGG pathways in RF vs. RY included Axon guidance, Fc epsilon RI signaling pathway, progesterone‐mediated oocyte maturation, and cell cycle (Figure 5a). Besides, several growth‐related pathways were also discovered in RF vs. RY, including Wnt signaling pathway, MAPK signaling pathway, and longevity regulating pathway. The candidate genes in ZF vs. ZY were highly enriched in non–small‐cell lung cancer, ovarian steroidogenesis, cortisol synthesis and secretion, and phagosome (Figure 5b). In addition, a few enriched pathways in ZF vs. ZY have known functions associated with growth regulation, such as PI3K‐Akt signaling pathway, p53 signaling pathway, Hippo signaling pathway, and cell cycle. More importantly, cell cycle and longevity regulating pathway were both enriched in RF vs. RY and ZF vs. ZY.

### 3.5 Function categories and schematic model for candidate genes

**FIGURE 6:**
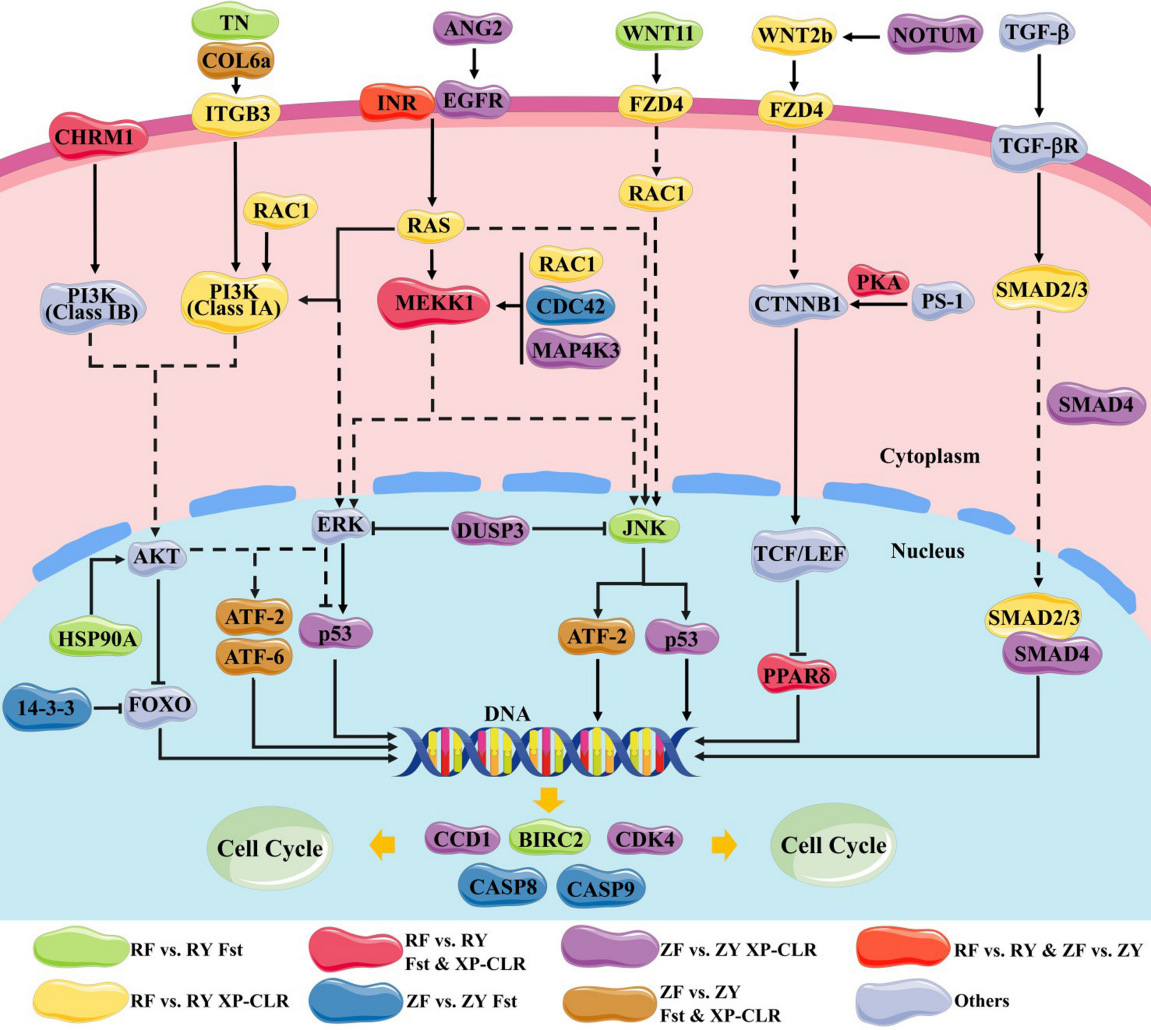
Schematic diagram of candidate genes from growth‐related pathways under artificial selection. Candidate genes were selected by *F*_st_, XP‐CLR, and both *F*_st_ and XP‐CLR in the comparison between fast‐growing RF strain and wild RY population were marked with green, yellow, and rose colors, respectively. In the comparison between fast‐growing ZF strain and wild ZY population, blue, purple, and brown colors were used to represent the candidate genes identified by *F*_st_, XP‐CLR, and both *F*_st_ and XP‐CLR, respectively. Candidate genes with selection in both comparisons were shown in red. Additionally, the components of signaling pathway, not selected, were colored gray. The detailed information of the candidate genes was listed in Table S8

Based on KEGG enrichment analysis, the candidate genes enriched in growth‐related pathways were further investigated. We proposed a schematic model of 33 candidate genes potentially under selection acting as key components of various signaling pathways with close connection, which further took part in regulating the process of cell cycle (Figure 6, Table S8). Specifically, several candidate genes were coding for extracellular matrix protein (tenascin‐R, TN; collagen type VI alpha, COL6a) and growth factors (angiopoietin 2, ANG2; protein Wnt‐2b‐A, WNT2b; wingless‐type MMTV integration site family, member 11, WNT11). Meanwhile, muscarinic acetylcholine receptor M1 (CHRM1), integrin beta pat‐3 (ITGB3), INR, epidermal growth factor receptor (EGFR), and frizzled 4 (FZD4) function as the key receptors for the extracellular matrix protein and growth factors. Phosphatidylinositol 4,5‐bisphosphate 3‐kinase catalytic subunit delta (PI3K), GTPase HRas (RAS), mitogen‐activated protein kinase kinase 1 (MEKK1), and ras‐related C3 botulinum toxin substrate 1 (RAC1) were key components of the signaling pathways with important roles in signaling cascades in cells. Additionally, cyclic AMP‐dependent transcription factor ATF2 (ATF2), cyclic AMP‐dependent transcription factor ATF‐6 (ATF‐6), cellular tumor antigen p53 (p53), and peroxisome proliferator‐activated receptor (PPAR) genes are implicated in mediating transcription of various genes. G1/S‐specific cyclin‐D1 (CCD1) and cell division control protein 42 (CDK4) genes play crucial roles in control of cell cycle, while caspase 8 (CASP8) and caspase 9 (CASP9) are involved in cell apoptosis. These candidate genes could have an influence on signaling transduction of growth and cell cycle, which were synergistically selected during generations of selective breeding.

## 4 DISCUSSION

Great improvement of growth has been achieved in the RF and ZF oyster strains as reported in the previous studies (Li et al., 2011; Wang & Li, 2017; Zhang et al., 2019), after generations of artificial selection. However, the way in which the oyster genome was shaped during selective breeding process remains unknown. In the present study, we performed whole‐genome resequencing of 40 oysters to profile the genetic variations of selective strains and wild populations, providing opportunities to inspect genetic and genomic differentiations during artificial selection. In total, 12.20 million high confidence SNPs were identified in Pacific oyster, which washigher than those in other oysters, such as 18,849 SNPs in Portuguese oyster (*Crassostrea angulata*) (Vu et al., 2021), 14,950 SNPs in European oyster (*Ostrea edulis*) (Vera et al., 2019), and 5,243 SNPs in black‐lip pearl oyster (*Pinctada margaritifera*) (Lal et al., 2016). Moreover, the average SNP density of Pacific oyster (20.71 SNPs/kb) is almost twice as many as that in high‐heterozygosity threespine stickleback (*Gasterosteus aculeatu*s) (11 SNPs/kb) with similar sequencing depths (Shanfelter et al., 2019). The elevated heterozygosity could provide a fitness advantage or possible resource for responses to natural selection (Warren et al., 2018). PCA and phylogenetic analysis provided strong evidence for the classification of different groups of oysters, which were consistent with the observation of population structure. Interestingly, great genetic differentiation was observed between RF and ZF groups, both of which have undergone the same artificial selection pressure and direction for 10 generations. For the LD decay, it was declined rapidly with increase in physical distance, which were associated with the high density of SNPs in oysters. Nevertheless, there still remained evidence of artificial selection, the long‐range LD, in both RF and ZF groups under selection.

The *F*_st_ and XP‐CLR have been widely recognized as the effective methods for identification of genomic signatures under selection, which are based on genetic differentiation and allele frequency difference, respectively (Barreiro et al., 2008; Chen et al., 2010). In the present study, we revealed numerous breeding signatures reflecting the complex genetic and genomic architectures from ten‐generation artificial selection of Pacific oysters. The genes identified from these genomic signatures under selection are closely associated with a few growth‐related pathways, such as cell cycle, longevity regulating pathway, Wnt signaling pathway, PI3K‐Akt signaling pathway, MAPK signaling pathway, and Hippo signaling pathway, which could be involved in growth regulation. Overall, these genes are known as key receptors, kinase, or regulators in these signaling pathways and play critical roles in the regulation of cell cycle.

Remarkably, growth factor (ANG2), extracellular matrix protein (COL6a and TN), and receptors (INR, EGFR, ITGB3, and CHRM1) in MAPK and PI3K‐Akt signaling pathways have been undergone selection during breeding process. As novel endothelial growth factors, ANG2 could bind to EGFR to regulate growth and development of blood vessel via MAPK and PI3K‐Akt signaling pathways (Niu & Carter, 2007). ITGB3 is a conserved receptor, which could recognize the extracellular matrix (ECM) components, such as COL6a and TN (Brown, 2011; Hynes, 1999). CHRM1 is involved in the activation of PI3K‐Akt signaling pathways (Cui et al., 2006; Zhao et al., 2019). INR gene was consistently detected by *F*_st_ and XP‐CLR methods in both comparisons, suggesting its strong selection pressure. In invertebrates, INR, a tyrosine kinase receptor, could interact with insulin‐related peptide and simulate MAPK and PI3K‐Akt signaling pathways to regulate the processes of growth, development, and metabolism (Shi et al., 2013; Zhang et al., 2017). Selection of these functional genes could impact the communication between extracellular and intracellular signals for cell growth and cycle. Furthermore, PI3K (class A), RAC1, RA, and MEKK1 genes, which act as key components in PI3K‐Akt and MAPK signaling pathways (Bökel & Brown, 2002; Howe et al., 1998), were also under selection and may affect signaling cascades in the cells. Wnt signaling pathway is widely known for its crucial roles in development (Cadigan & Nusse, 1997; Wodarz & Nusse, 1998). The WNT signaling transduction to TCF or JNK signaling pathway would be influenced by selection of WNT11, WNT2b, and their seven‐transmembrane‐type receptor FZD4 (Boutros et al., 1998; Kühl et al., 2000; Sagara et al., 2001).

The selective sweeps were also observed to contain the downstream transcription factors, including ATF2, ATF‐6, p53, and PPAR. ATF2 and ATF‐6 are members of the ATF/cAMP response element‐binding (CREB) protein family (Hai & Hartman, 2001), which are usually modulated by extracellular signals and required for regulating transcription of genes associated with cell cycle such as CDK4 and CCD1 (Andrisani, 1999; Beier et al., 1999; Cesare et al., 1999; Xiong et al., 2017). To be noted, p53 is implicated in cell cycle arrest as a trans‐activator that works to negatively regulate a set of genes essential for this process (Chen, 2016; Stewart & Pietenpol, 2001). So far, three PPAR isoforms, α, β/δ, and γ, have been identified in vertebrates, while only one PPAR homolog was identified in Pacific oyster (Evans et al., 2004; Vogeler et al., 2014). PPARs are known as ligand‐activated nuclear receptors that play important roles in controlling lipid and glucose homeostasis in distinct tissues (Zhou et al., 2013). Additionally, PPAR is also involved in cell proliferation, death, and differentiation by controlling a number of cell cycle genes, such as cyclin A, cyclin D (CCD), cyclin‐dependent kinase 2, and CDK4 (Vignati et al., 2006; Zaveri et al., 2009). Obviously, selection of these transcription factors would have impacted on regulation of the transcriptional response of the key genes related to cell cycle. In addition, it was found that there was obvious evidence of selection in the central mediators of TGF‐β signaling pathway, including SMAD2 and SMAD4, which are also associated with the regulation of cell cycle proteins at transcription level (Chae et al., 2017; Kretschmer et al., 2003). The CCD1, CKD4, and BIRC2 have been well documented as the modulators of cell cycle (Baldin et al., 1993; Tam et al., 1994; Yang & Li, 2000), while CASP8 and CASP9 worked as the molecular switches for cell apoptosis (Hakem et al., 1998; Kuwana et al., 1998). The selective sweeps located near the modulators of cell cycles or molecular switches could provide direct effects on cell cycle. There is substantial evidence of a relationship between growth and cell cycle that growth is mediated largely by cell cycle (Beemster et al., 2003; Edgar, 1998). Thus, the candidate genes under selection were involved in various signaling pathways with close connection and further participated in regulating the process of cell cycle, which would make a great contribution to the fast‐growing trait in RF and ZF strains of Pacific oyster. However, the specific effects of artificial selection on the physiological function of the candidate genes required further investigations.

Direct comparison between RF and ZF revealed the great divergence of the fast‐growing strains, which have undergone the same selective pressure. Surprisingly, only small parts of genomic regions were commonly selected in both fast‐growing strains. Besides selection, genetic drift is another important factor to alter allele frequency. More importantly, the changes caused by genetic drift are random, not driven by environmental or adaptive pressure (Lynch et al., 2016; Masel, 2011). Genetic drift may be involved in the generations of genetic differences between RF and ZF fast‐growing strains, while whose genetic mechanism deserves further investigations.

## 5 CONCLUSION

In the present study, we performed whole‐genome resequencing of Pacific oysters to profile genetic variations and differentiations between two selective strains (RF and ZF) and wild populations (RY and ZY) during generations of artificial selection. The results revealed that 768 and 664 selective sweeps, containing 1042 and 872 genes, were related to fast‐growing trait of oysters in selective RF and ZF strains, respectively. Among them, numerous candidate genes act as key components of various signaling pathways with close connection and further take part in regulating the process of cell cycle, which could be responsible for the growth difference between selective strains and wild populations. This study not only enhances the understanding of genomic signatures associated with fast‐growing trait of selectively bred Pacific oysters, but also provides useful guidance for implementing further genomic research and breeding applications in Pacific oyster.

## CONFLICT OF INTEREST

The authors declared that the research was not any potential conflict of interest.

## Supporting information

Figures S1‐S3

Tables S1‐S3

Table S4

Table S5

Table S6

Table S7

Table S8

## Data Availability

All genomic sequence data used in this study have been deposited in the Sequence Read Archive (SRA) of National Center for Biotechnology Information with the BioProject accession number of PRJNA543621.
