# Supplementary material for: Genomic signatures of artificial selection in the Pacific oyster, Crassostrea gigas
Source: Evol Appl. 2021 Sep 2;15(4):618–30. doi: 10.1111/eva.13286 (PMC9046764; doi:10.1111/eva.13286)
Supplement: Supplementary file 1 — Figures S1‐S3 [file EVA-15-618-s006.doc]

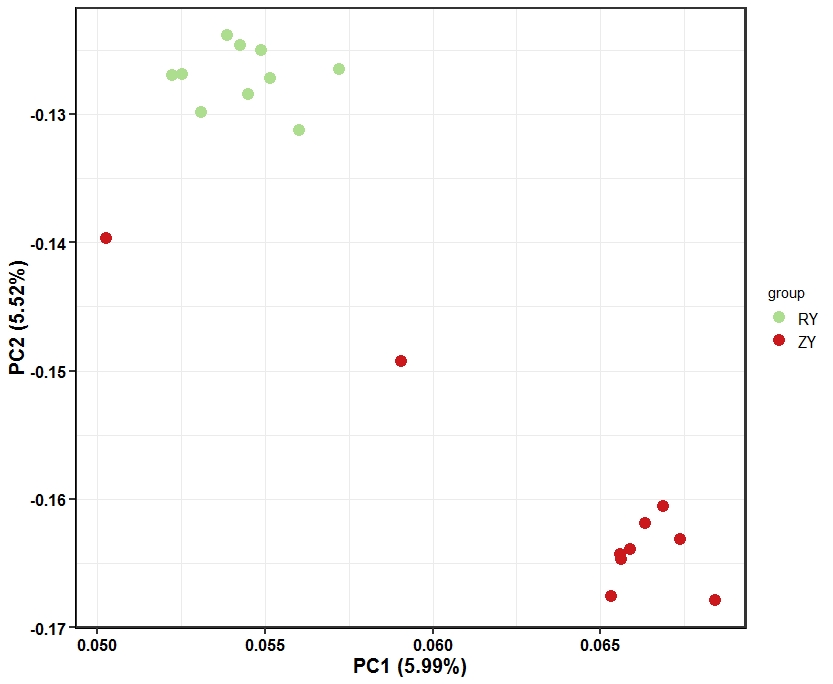


**Figure S1** Principal component analysis (PCA) of the top two components (PC1 and PC2) of RY and ZY populations.


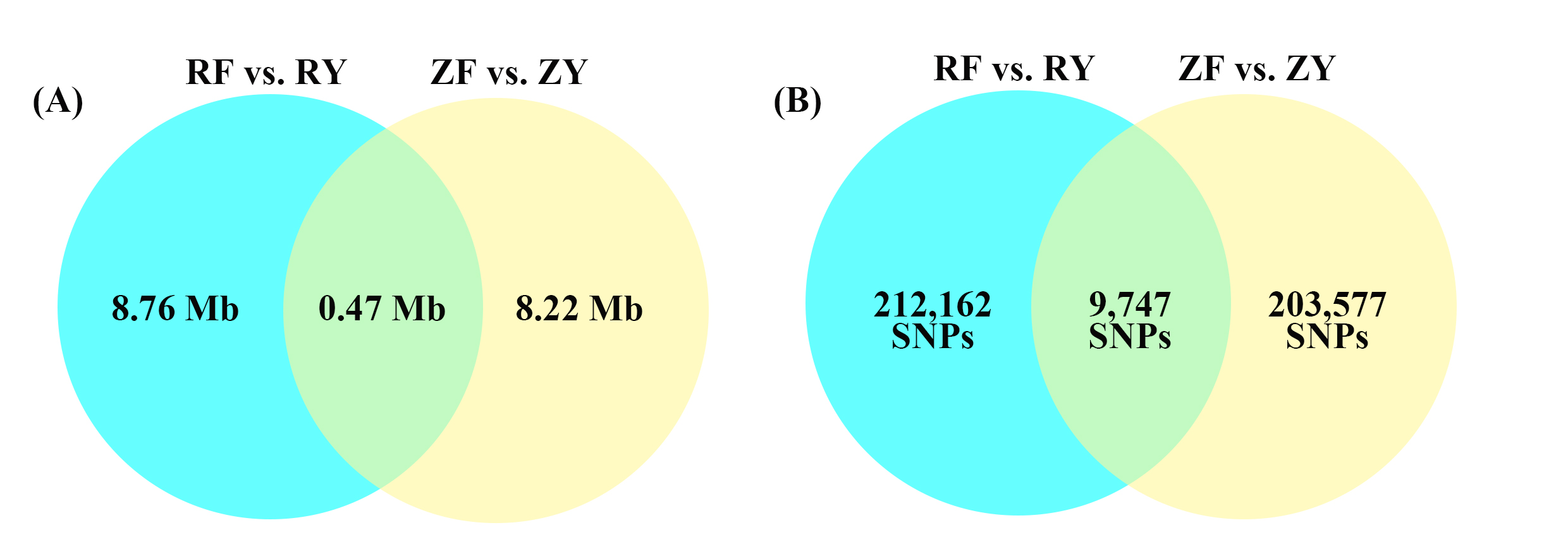


**Figure S2** Venn diagram of genomic regions (A) and SNPs (B) associated with selective sweeps in RF vs. RY and ZF vs. ZY.


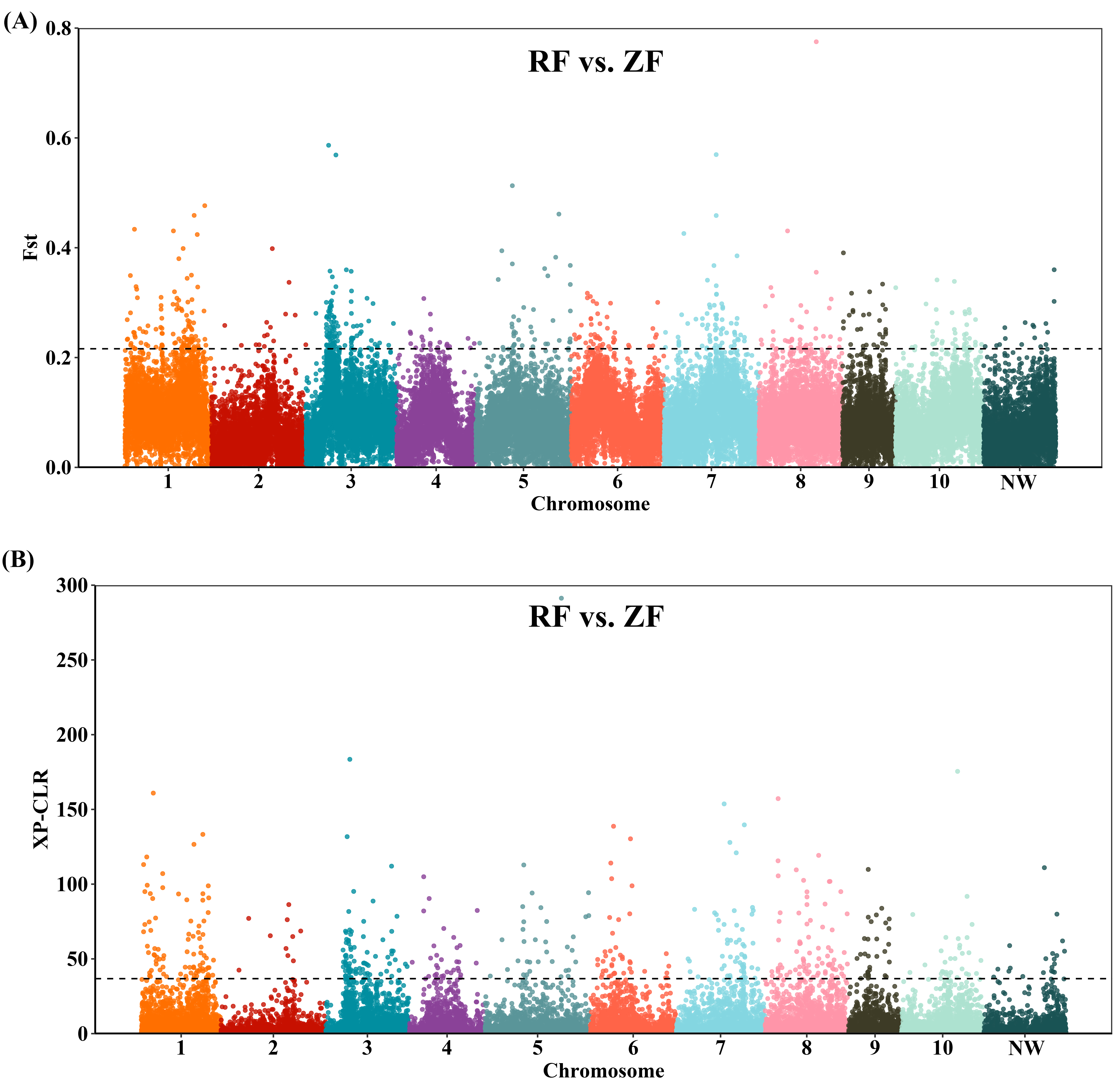


**Figure S3** Genome-wide distribution of selective sweeps in RF vs. ZF fast-growing strains. (A) Selective sweeps in RF vs. ZF were identified by Fst. The horizontal dash line represented the top 1% threshold in Fst value (0.22). (B) Selective sweeps in RF vs. ZF were identified by XP-CLR. The horizontal dash line represented the top 1% threshold in XP-CLR scores (36.77).
