# Supplementary material for: Genomic signatures of artificial selection in the Pacific oyster, Crassostrea gigas
Source: Evol Appl. 2021 Sep 2;15(4):618–30. doi: 10.1111/eva.13286 (PMC9046764; doi:10.1111/eva.13286)
Supplement: Supplementary file 2 — Tables S1‐S3 [file EVA-15-618-s004.doc]

**Table S1** Whole genome re-sequencing data of 40 Pacific oysters.

| **Sample ID** | **Raw_base** | **Clean_base** | **Effective Rate_base** | **Raw_reads** | **Clean_reads** | **Effective Rate_reads** | **Q20** | **Q30** | **Coverage_10X** | **mapped reads** | **Unique mapped reads** |
| --- | --- | --- | --- | --- | --- | --- | --- | --- | --- | --- | --- |
| ZY_1 | 5,944,655,700 | 5,820,156,273 | 97.90% | 39,631,038 | 38,959,072 | 98.30% | 97.22% | 92.34% | 8.98 | 38,108,801 | 27,210,662 |
| ZY_3 | 6,410,923,200 | 6,275,209,065 | 97.88% | 42,739,488 | 41,999,404 | 98.26% | 97.31% | 92.53% | 9.68 | 40,864,108 | 29,196,179 |
| ZY_4 | 7,023,851,700 | 6,879,157,448 | 97.94% | 46,825,678 | 46,033,174 | 98.30% | 97.28% | 92.43% | 10.61 | 45,013,163 | 32,217,543 |
| ZY_5 | 5,952,304,500 | 5,829,017,638 | 97.92% | 39,682,030 | 39,040,262 | 98.38% | 97.37% | 92.65% | 8.99 | 38,144,717 | 27,198,878 |
| ZY_6 | 6,652,179,900 | 6,506,432,588 | 97.80% | 44,347,866 | 43,539,528 | 98.17% | 97.10% | 92.04% | 10.04 | 42,533,350 | 30,434,635 |
| ZY_7 | 6,793,414,800 | 6,634,375,845 | 97.65% | 45,289,432 | 44,533,918 | 98.33% | 97.46% | 92.86% | 10.23 | 43,724,087 | 31,054,842 |
| ZY_16 | 6,530,791,800 | 6,396,242,118 | 97.93% | 43,538,612 | 42,811,292 | 98.32% | 97.37% | 92.74% | 9.87 | 41,687,495 | 29,777,825 |
| ZY_19 | 6,045,315,900 | 5,924,612,111 | 98.00% | 40,302,106 | 39,653,414 | 98.39% | 97.46% | 92.83% | 9.14 | 38,721,860 | 27,804,307 |
| ZY_20 | 7,542,661,800 | 7,382,219,394 | 97.87% | 50,284,412 | 49,364,094 | 98.16% | 97.62% | 93.23% | 11.39 | 47,743,586 | 34,409,662 |
| ZY_24 | 6,119,298,300 | 5,975,092,216 | 97.64% | 40,795,322 | 39,980,416 | 98.00% | 97.47% | 92.87% | 9.22 | 38,975,185 | 27,897,156 |
| ZF10_1 | 6,776,166,000 | 6,605,702,902 | 97.48% | 45,174,440 | 44,168,978 | 97.77% | 97.27% | 92.42% | 10.19 | 42,669,375 | 30,524,334 |
| ZF10_2 | 6,686,622,300 | 6,547,441,310 | 97.91% | 44,577,482 | 43,780,054 | 98.21% | 97.64% | 93.26% | 10.10 | 42,137,678 | 30,220,475 |
| ZF10_3 | 7,203,695,400 | 7,017,029,675 | 97.40% | 48,024,636 | 46,928,288 | 97.71% | 97.05% | 91.83% | 10.83 | 45,312,049 | 32,568,155 |
| ZF10_5 | 6,589,753,500 | 6,434647,782 | 97.64% | 43,931,690 | 43,028,432 | 97.94% | 97.40% | 92.69% | 9.93 | 41,699,276 | 29,879,639 |
| ZF10_6 | 7,485,608,100 | 7,301,975,663 | 97.54% | 49,904,054 | 48,802,360 | 97.79% | 95.99% | 89.54% | 11.27 | 47,343,854 | 33,912,235 |
| ZF10_10 | 6,014,678,700 | 5,873,117,138 | 97.64% | 40,097,858 | 39,287,016 | 97.97% | 97.41% | 92.70% | 9.06 | 37,930,129 | 27,275,953 |
| ZF10_11 | 6,314,001,900 | 6,168,686,441 | 97.69% | 42,093,346 | 41,262,054 | 98.02% | 97.42% | 92.72% | 9.52 | 39,968,886 | 28,680,562 |
| ZF10_12 | 6,570,139,200 | 6,417,133,928 | 97.67% | 43,800,928 | 42,906,060 | 97.95% | 97.50% | 92.97% | 9.90 | 41,487,043 | 29,818,953 |
| ZF10_13 | 6,415,911900 | 6,249,886,201 | 97.41% | 42,772,746 | 41,810,302 | 97.74% | 97.14% | 92.02% | 9.64 | 40,339,098 | 28,916,413 |
| ZF10_14 | 7,583462100 | 7,431,699,603 | 97.99% | 50,556,414 | 49702,286 | 98.31% | 97.76% | 93.60% | 11.47 | 47,946,190 | 34,161,033 |
| RY_11 | 6,013493400 | 5,879,377,670 | 97.76% | 40,089,956 | 39,316,386 | 98.07% | 97.37% | 92.59% | 9.07 | 38,210,739 | 27,608,804 |
| RY_13 | 6,248,988,300 | 6,110,280,682 | 97.78% | 41,659,922 | 40,868,774 | 98.10% | 97.53% | 92.93% | 9.43 | 39,692,614 | 28,746,148 |
| RY_15 | 6,232,500,900 | 6,104,793,789 | 97.95% | 41,550,006 | 40,844146 | 98.30% | 97.26% | 92.42% | 9.42 | 39,526,627 | 28,627,675 |
| RY_24 | 6,663,912,600 | 6,501,505,705 | 97.56% | 44,426,084 | 43,464,522 | 97.83% | 96.82% | 91.43% | 10.03 | 41,959,412 | 30,352,608 |
| RY_25 | 6,436,725,900 | 6,286,403,089 | 97.66% | 42,911,506 | 42,050,538 | 97.99% | 97.37% | 92.59% | 9.70 | 40,732,420 | 29,352,190 |
| RY_29 | 7,542,062,100 | 7,381,866,375 | 97.87% | 50,280,414 | 49,358,030 | 98.16% | 97.32% | 92.53% | 11.39 | 47,914,432 | 34,779,138 |
| RY_31 | 6,576,510,000 | 6,447,226,819 | 98.03% | 43,843,400 | 43,175,788 | 98.47% | 97.50% | 93.02% | 9.95 | 42,114,386 | 30,377,968 |
| RY_34 | 8,469,132,600 | 8,278,685,678 | 97.75% | 56,460,884 | 55,353,416 | 98.03% | 97.22% | 92.24% | 12.77 | 53,818,898 | 38,701,008 |
| RY_35 | 7,375,122,600 | 7,201,833,251 | 97.65% | 49,167,484 | 48,158,288 | 97.94% | 97.09% | 91.92% | 11.11 | 46,667,501 | 33,719,094 |
| RY_39 | 9,056,322,000 | 8,821,787,273 | 97.41% | 60,375,480 | 58,966822 | 97.66% | 97.00% | 91.74% | 13.61 | 57,040,837 | 41,005,476 |
| RF10_1 | 8,569,927,800 | 8,350,422,241 | 97.43% | 57,132,852 | 55,879,086 | 97.80% | 97.13% | 92.07% | 12.88 | 53,999,765 | 38,309,159 |
| RF10_2 | 6,629,953,800 | 6,497,643,693 | 98.00% | 44,199,692 | 43,478,162 | 98.36% | 97.37% | 92.72% | 10.02 | 42,023,297 | 30,062,117 |
| RF10_3 | 6,860353,200 | 6,732,966,447 | 98.14% | 45,735,688 | 45,076,210 | 98.55% | 97.55% | 93.07% | 10.39 | 44,139,247 | 31,638,423 |
| RF10_4 | 6,912,348,600 | 6,785,932,586 | 98.17% | 46,082,324 | 45,438,214 | 98.60% | 98.10% | 94.36% | 10.47 | 44,599,203 | 31,971,404 |
| RF10_5 | 6,693,003,600 | 6,572,598,059 | 98.20% | 44,620,024 | 43,991,862 | 98.59% | 97.56% | 93.06% | 10.14 | 43,078,309 | 30,852,692 |
| RF10_6 | 6,837,001,800 | 6,702,750,317 | 98.03% | 45,580,012 | 44,853688 | 98.40% | 97.41% | 92.77% | 10.34 | 43,621,342 | 31,306,438 |
| RF10_8 | 6,014,920,500 | 5,885,285,044 | 97.84% | 40,099,470 | 39,390,174 | 98.23% | 97.18% | 92.21% | 9.08 | 38,544,827 | 27,791,359 |
| RF10_9 | 6,885,295,800 | 6,742,554,958 | 97.92% | 45,901,972 | 45,101,418 | 98.25% | 97.33% | 92.62% | 10.40 | 43,943,002 | 31,510,397 |
| RF10_10 | 6,603,879,300 | 6,468,254,006 | 97.94% | 44,025,862 | 43,258,350 | 98.25% | 97.69% | 93.41% | 9.98 | 42,091,086 | 30,323,058 |
| RF10_12 | 6,756,033,000 | 6,609,362,093 | 97.82% | 45,040,220 | 44,224,890 | 98.18% | 97.26% | 92.48% | 10.20 | 42,903,055 | 30,794,251 |

**Table S2** Summary of SNPs information.

|  | Oyster (*C. gigas*) |
| --- | --- |
| Sample size | 40 |
| SNP | 12,208,101 |
| 5’ UTR | 167,532 (1.37%) |
| 3’ UTR | 561,456 (4.60%) |
| Upstream^a^ | 1,709,581 (14.00%) |
| Downstream^b^ | 433,992 (3.56%) |
| Intergenic | 2,557,745 (20.95%) |
| Intronic | 5,498,655 (45.04%) |
| Exonic | 1,266,880 (10.38%) |
| Others | 12,260 (0.10%) |
| Synonymous | 773,649 (61.07%) |
| Non-synonymous | 493,231(38.93%) |
| Non-synonymous/Synonymous | 0.64 |

^ab^Upstream and downstream represented the 5kb upstream and downstream of genes, respectively.

**Table S3 Distribution of SNPs across Pacific oyster genome.**

| Chromosome | Length | SNPs | Density (SNPs/kb) |
| --- | --- | --- | --- |
| LG1 | 55,785,328 | 1,390,995 | 24.93 |
| LG2 | 73,222,313 | 862,009 | 11.77 |
| LG3 | 58,319,100 | 1,438,509 | 24.67 |
| LG4 | 53,127,865 | 1,140,008 | 21.46 |
| LG5 | 73,550,375 | 1,124,855 | 15.29 |
| LG6 | 60,151,564 | 1,494,462 | 24.85 |
| LG7 | 62,107,823 | 1,523,035 | 24.52 |
| LG8 | 58,462,999 | 1,131,925 | 19.36 |
| LG9 | 37,089,910 | 726,187 | 19.58 |
| LG10 | 57,541,580 | 1,376,116 | 23.92 |
| Total | 589,358,857 | 12,208,101 | 20.71 |
