# Supplementary material for: Genomic signatures of artificial selection in the Pacific oyster, Crassostrea gigas
Source: Evol Appl. 2021 Sep 2;15(4):618–30. doi: 10.1111/eva.13286 (PMC9046764; doi:10.1111/eva.13286)
Supplement: Supplementary file 7 — Table S8 [file EVA-15-618-s001.doc]

**Table S8** Full name of genes involved in various signaling pathway and cell cycle.

| Abbreviation | Full name | KO | Gene id | RF vs. RY | Fst | XPCLR | ZF vs. ZY | Fst | XP-CLR |
| --- | --- | --- | --- | --- | --- | --- | --- | --- | --- |
| AKT | AKT serine/threonine kinase | K04456 | - | - | - | - | - | - | - |
| ANG2 | angiopoietin 2 | K05466 | LOC105334700 | - | - | - | √ | - | √ |
| ATF-2 | cyclic AMP-dependent transcription factor ATF-2 | K04450 | LOC105323178 | - | - | - | √ | √ | √ |
| ATF-6 | cyclic AMP-dependent transcription factor ATF-6 | K09049 | LOC105326811 | - | - | - | √ | √ | √ |
| BIRC2 | baculoviral IAP repeat containing 2 | K16060 | LOC117692267 | √ | √ | - | - | - | - |
| CASP8 | Caspase 8 | K04398 | LOC117687949 | - | - | - | √ | √ | - |
| CASP9 | Caspase 9 | K04399 | LOC105325340 | - | - | - | √ | √ | - |
| CCD1 | G1/S-specific cyclin-D1 | K04503 | LOC105330764 | - | - | - | √ | - | √ |
| CDC42 | cell division control protein 42 | K04393 | LOC105341297 | - | - | - | √ | √ | - |
| CDK4 | cyclin-dependent kinase 4 | K02089 | LOC105327718 | - | - | - | √ | - | √ |
| CHRM1 | muscarinic acetylcholine receptor M1 | K04129 | LOC105327246 | √ | √ | √ | - | - | - |
| COL6a | collagen type VI alpha | K06238 | LOC105319934 | - | - | - | √ | √ | √ |
| CTNNB1 | catenin beta 1 | K02105 | - | - | - | - | - | - | - |
| DUSP3 | dual specificity protein phosphatase 3 | K17614 | LOC105334705 | - | - | - | √ | - | √ |
| EGFR | epidermal growth factor receptor | K04361 | LOC105320592 | - | - | - | √ | - | √ |
| ERK | mitogen-activated protein kinase 1/3 | K04371 | - | - | - | - | - | - | - |
| FOXO | forkhead box protein O_3_ | K09408 | - | - | - | -- |  | - | - |
| FZD4 | Frizzled 4 | K02354 | LOC117681348 | √ | - | √ | - | - | - |
| HSP90A | heat shock protein 90 alpha family class A member 1 | K04079 | LOC105345989 | √ | √ | - | - | - | - |
| ITGB3 | integrin beta pat-3 | K06493 | LOC105332751 | √ | - | √ | - | - | - |
| INR | insulin-like peptide receptor | K04527 | LOC105348544 | √ | √ | √ | √ | √ | √ |
| JNK | mitogen-activated protein kinase 8 | K04440 | LOC105338795 | √ | √ | - | - | - | - |
| LEF | lymphoid enhancer binding factor 1 | K04492 | - | - | - | - | - | - | - |
| MAP4K3 | mitogen-activated protein kinase kinase kinase kinase 3 | K04406 | LOC105341571 | - | - | - | √ | - | √ |
| MEKK1 | mitogen-activated protein kinase kinase 1 | K04416 | LOC105331404 | √ | √ | √ | - | - | - |
| NOTUM | O-palmitoleoyl-L-serine hydrolase | K19882 | LOC105330154 | - | - | - | √ | - | √ |
| PI3K | phosphatidylinositol 4,5-bisphosphate 3-kinase catalytic subunit delta | K00922 | LOC105320169 | √ | - | √ | - | - | - |
| PKA | cAMP-dependent protein kinase catalytic subunit 1 | K04345 | LOC105336635 | √ | √ | √ | - | - | - |
| PPAR | peroxisome proliferator-activated receptor | K04504 | LOC105323212 | √ | √ | √ | - | - | - |
| PS-1 | presenilin 1 | K04505 | - | - | - | - | - | - | - |
| p53 | cellular tumor antigen p53 | K04451 | LOC105340434 | - | - | - | √ | - | √ |
| RAC1 | ras-related C3 botulinum toxin substrate 1 | K04392 | LOC105344224 | √ | - | √ | - | - | - |
| RAS | GTPase HRas | K07827 | LOC105342014 | √ | - | √ | - | - | - |
| SMAD2 | mothers against decapentaplegic homolog 2 | K04500 | LOC105337701 | √ | - | √ | - | - | - |
| SMAD3 | mothers against decapentaplegic homolog 3 | K23605 | - | - | - | - | - | - | - |
| SMAD4 | mothers against decapentaplegic homolog 4 | K04501 | LOC105331371 | - | - | - | √ | - | √ |
| TCF | transcription factor 7 | K02620 | - | - | - | - | - | - | - |
| TN | tenascin-R | K06252 | LOC105345860 | √ | √ | - | - | - | - |
| WNT2b | protein Wnt-2b-A | K00182 | LOC105340175 | √ | - | √ | - | - | - |
| WNT11 | wingless-type MMTV integration site family, member 11 | K01384 | LOC105322578 | √ | √ | - | - | - | - |
| 14-3-3 | 14-3-3 protein beta/theta/zeta | K16197 | LOC105337335 | - | - | - | √ | √ | - |
